# Analyzing the Mechanisms Behind Macrolide Antibiotic-Induced Liver Injury Using Quantitative Systems Toxicology Modeling

**DOI:** 10.1007/s11095-019-2582-y

**Published:** 2019-02-07

**Authors:** Jeffrey L. Woodhead, Kyunghee Yang, David Oldach, Chris MacLauchlin, Prabhavathi Fernandes, Paul B. Watkins, Scott Q. Siler, Brett A. Howell

**Affiliations:** 1 DILIsym Services, Inc., a Simulations Plus Company, 6 Davis Drive, PO Box 12317, Research Triangle Park, North Carolina 27709 USA; 2 Cempra, Inc., Chapel Hill, North Carolina USA; 3 UNC Eshelman School of Pharmacy, The University of North Carolina at Chapel Hill, Chapel Hill, North Carolina USA

**Keywords:** antibiotics, BSEP inhibition, liver injury, mitochondria, quantitative systems toxicology

## Abstract

**Purpose:**

Macrolide antibiotics are commonly prescribed treatments for drug-resistant bacterial infections; however, many macrolides have been shown to cause liver enzyme elevations and one macrolide, telithromycin, has been pulled from the market by its provider due to liver toxicity. This work seeks to assess the mechanisms responsible for the toxicity of macrolide antibiotics.

**Methods:**

Five macrolides were assessed in *in vitro* systems designed to test for bile acid transporter inhibition, mitochondrial dysfunction, and oxidative stress. The macrolides were then represented in DILIsym, a quantitative systems pharmacology (QST) model of drug-induced liver injury, placing the *in vitro* results in context with each compound’s predicted liver exposure and known biochemistry.

**Results:**

DILIsym results suggest that solithromycin and clarithromycin toxicity is primarily due to inhibition of the mitochondrial electron transport chain (ETC) while erythromycin toxicity is primarily due to bile acid transporter inhibition. Telithromycin and azithromycin toxicity was not predicted by DILIsym and may be caused by mechanisms not currently incorporated into DILIsym or by unknown metabolite effects.

**Conclusions:**

The mechanisms responsible for toxicity can be significantly different within a class of drugs, despite the structural similarity among the drugs. QST modeling can provide valuable insight into the nature of these mechanistic differences.

**Electronic supplementary material:**

The online version of this article (10.1007/s11095-019-2582-y) contains supplementary material, which is available to authorized users.

## Introduction

The macrolide class of antibiotics are frequently prescribed antibiotics for otherwise drug-resistant bacterial infections (1,2). Several macrolide antibiotics are currently available for clinical use. Erythromycin is the first generation macrolide; it was isolated from the bacteria *Saccharopolyspora erythraea* in the 1950s. Clarithromycin and azithromycin are the second generation macrolides; they are semi-synthetic derivatives of erythromycin. Erythromycin, clarithromycin, and azithromycin have been reported to cause mild, asymptomatic elevations in serum alanine aminotransferase (ALT) in 1–2% of the population (3), and are associated with very rare cases of clinically important liver injury (4). Widespread resistance to existing macrolides necessitated development of the next generation of new antibiotics. Telithromycin is a macrolide where the cladinose sugar found in the older macrolides is replaced with a keto group in addition to other changes, thus called a ketolide. Telithromycin showed activity against macrolide-resistant strains and was approved by regulatory agencies for marketing in the early 2000s. However, rare cases of serious liver injury including acute liver failure occurred in patients treated with telithromycin (5), which led to a boxed warning about serious liver toxicity and restriction of indication. As a result, telithromycin was voluntarily pulled from the U.S. market by its provider (6,7). After telithromycin, no other macrolide antibiotics have been approved for clinical use in the U.S.

Solithromycin, a novel macrolide antibiotic and the first fluoroketolide, has been developed to treat moderate to moderately-severe community-acquired bacterial pneumonia (CABP) and otherwise drug-resistant bacterial infections. In multi-center phase III clinical trials with CABP patients, solithromycin showed promise by proving non-inferiority to moxifloxacin (8,9). However, serum ALT elevations occurred with solithromycin at a higher frequency than with other macrolide antibiotics; in phase III clinical trials, 5% and 9% of patients developed benign ALT elevations above the 3-fold upper limit of normal (ULN) with the Oral and the IV-to-Oral protocols, respectively (8,9). Furthermore, solithromycin is structurally similar to telithromycin in also being a ketolide (see Fig. C1 in the supplemental materials), further raising concerns about solithromycin’s liver safety profile (10). The FDA has demanded greatly expanded clinical trials of solithromycin to further assess liver safety prior to an approval decision (11).

**Fig. 1:**
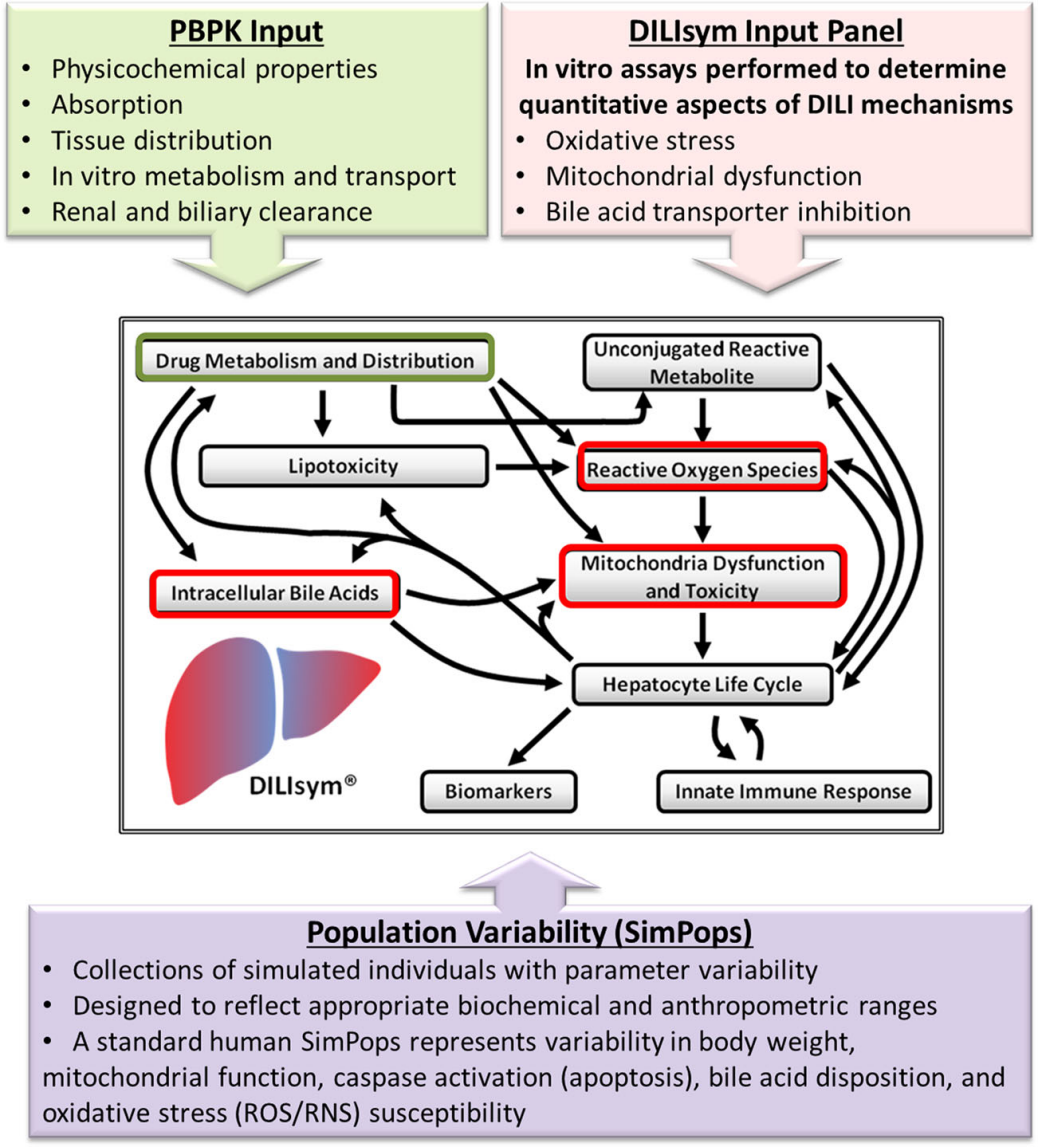
Quantitative systems toxicology modeling process using DILIsym.

Quantitative systems toxicology (QST) is a discipline of pharmacology that seeks to understand and ultimately predict the toxic effects of drugs/chemicals by integrating computational and experimental methods (12). DILIsym is a QST model of liver injury which integrates the results from *in vitro* mechanistic toxicity assays with estimates of *in vivo* exposure and known biochemistry to understand hepatotoxicity and the biochemical processes behind it (Fig. 1) (13–15). Hepatotoxicity mechanisms represented in DILIsym include oxidative stress, mitochondrial dysfunction, and bile acid transport inhibition, which are mechanistically connected to cell death and ALT elevation through previously described representations of liver biochemistry and physiology (13,14,16–18). Through these mechanisms, DILIsym has successfully predicted hepatotoxic potential of drugs and drug candidates and determined the underlying mechanisms of clinically observed hepatotoxicity signals (16,17,19). In the current study, DILIsym was used to determine the most likely mechanisms behind the ALT elevations observed with five macrolide antibiotics: solithromycin, erythromycin, clarithromycin, telithromycin, and azithromycin. Understanding the mechanisms behind the ALT elevations observed within this drug class could be important in determining whether novel antibiotics might have the same liver safety concerns that scuttled telithromycin.

## Materials and Methods

### Software Platform

DILIsym v5A was used to conduct the simulations in this paper. DILIsym is a software package that is available to members of the DILI-sim Initiative; academic and regulatory licensing is also available.

### Development of Physiologically-Based Pharmacokinetic (PBPK) Models

PBPK models for the five macrolide antibiotics were constructed within DILIsym to describe disposition of macrolides in humans. For solithromycin, the PBPK model was based on plasma concentration-time data from clinical trials; literature reports of plasma time courses were used for azithromycin, telithromycin, erythromycin, and clarithromycin. The basic structure of the DILIsym PBPK sub-model has been discussed elsewhere (13,17,19,20); details and results of the PBPK modeling for the five macrolides are provided in Supplement A.

### ***In Vitro*** Mechanistic Toxicity Assays

Five macrolides were assessed in *in vitro* assays for three main hepatotoxicity mechanisms represented within DILIsym: mitochondrial dysfunction, oxidative stress, and bile acid transporter inhibition. To detect potential mitochondrial dysfunction signals, cellular respiration assays were conducted using a Seahorse XFe96 Flux Analyzer in HepG2 cells incubated with various concentrations of macrolides for 1 or 24 h. HepG2 cells were chosen in part because of their metabolic incompetence, since any effect observed in the HepG2 system can be attributed solely to the activity of the parent compound. Induction of oxidative stress was determined by high content screening using a fluorescent probe, dihydroethidium (DHE), in HepG2 cells incubated with various concentrations of macrolides for 1 or 24 h. In these whole cell-based assays, intracellular concentrations of macrolides were determined by LC/MS/MS analysis in parallel HepG2 cultures. Parameter values for macrolide mediated induction of mitochondrial dysfunction and oxidative stress were determined by reproducing the cellular respiration data and the oxidative stress data directly within DILIsym using measured intracellular concentrations. Inhibitory effects of macrolides for bile acid transporters were assessed experimentally using membrane vesicles overexpressing a bile acid efflux transporter (i.e., BSEP, MRP3, or MRP4) and CHO cells overexpressing NTCP or obtained from published literature. Detailed experimental methods are described in Supplement B. Mitochondrial dysfunction and oxidative stress assays were performed by Cyprotex, Inc. (Macclesfield, UK). Transporter inhibition assays were performed by Solvo Biotechnology (Budaors, Hungary).

### Translation into DILIsym Parameters

For each of the assays conducted, the results were translated into DILIsym parameters for use in the simulations. The method used for this translation was consistent across compounds. For the bile acid transporter parameters, the IC_50_ was used directly as the inhibition constant. Mode of inhibition was assumed to be mixed inhibition with α = 5. While competitive and non-competitive inhibition types may result in low and high extremes of potential bile acid accumulation, respectively, mixed inhibition with α = 5 leads to a median impact on bile acid accumulation. In addition, mixed inhibitors are more common compared to pure competitive or noncompetitive inhibitors. For mitochondrial dysfunction, the assay results comparing intracellular concentrations and OCR were recapitulated in MITOsym if the OCR decline was non-saturable and in DILIsym if the OCR decline was saturable; the resulting parameters were translated into DILIsym parameters using translation factors involving exemplar compounds, a process which has been reported elsewhere (18). For oxidative stress, the assay results were reproduced using DILIsym by mimicking *in vitro* conditions; appropriate parameter values for the oxidative stress effects were identified by comparing simulation results with the measured data.

### Simulations Conducted

DILIsym v5A was used to perform simulations on each of the antibiotics at their maximum recommended doses and typical duration of treatment. The protocols used for each drug were as follows:

- Solithromycin Oral protocol: PO 800 mg QD on day 1, and 400 mg QD on days 2–5
- Solithromycin IV-to-Oral protocol: 60-min IV infusion 400 mg QD on days 1–3, PO 800 mg QD on day 4, and PO 400 mg QD on days 5–7
- Erythromycin: PO 500 mg QD, 7 days
- Clarithromycin: PO 500 mg BID, 7 days
- Telithromycin: PO 800 mg QD, 10 days
- Azithromycin: PO 500 mg QD, 7 days

For each of the five macrolide antibiotics, the following simulation types were run:

- **SimPops simulations**: These simulations were conducted using the Human_ROS_apop_mito_BA_v4A_1 SimPops (*n* = 285) included in DILIsym v5A. This SimPops represents variability in parameters related to bile acid homeostasis, mitochondrial function, oxidative stress, apoptosis, and regeneration. A list of parameters varied in the v4A_1 SimPops, as well as the sources used in the construction of the SimPops, are shown in Table I.
- **Mechanistic Investigation simulations:** These simulations were conducted on a subset of simulated individuals found to be most susceptible to the toxicity from each antibiotic. Then one of the three mechanisms is inactivated for each of the Mechanistic Investigation simulations while the other mechanisms remain active; if inactivating the mechanism leads to a decrease in the number of simulated individuals in which ALT elevations occur, the mechanism is determined to be contributing to the toxicity; the relative magnitude of the decrease in ALT elevation frequency represents the relative contribution of each mechanism to the overall simulated toxicity (16).

**Table I:** A List of the Parameters Varied in the v4A_1 SimPops Included in DILIsym v5A

Data used to define parameter distributions (if applicable)		
Parameter symbol in DILIsym®	Parameter name in DILIsym®	Data source for distribution
ATP_decr_necrosis_Vmax	ATP decrement necrosis Vmax	Assumed standard deviation of ±20% and parameter range of 2.5 times the S.D. and validated with outcome data
Body_mass	Body Mass	Parameter range from NHANES III (human data)
GSH_pre_trans_Vmax	GSH precursor transport Vmax	Parameter range derived from (21)
GSHo	GSH basal level	Parameter range from (22,23)
HGF_prod_LSEC_Vmax	Maximum LSEC HGF production rate per liver LSEC	Assumed standard deviation of ±20% and parameter range of 2.5 times the S.D. and validated with outcome data
HGF_regen_Vmax	HGF mediated regeneration Vmax	Assumed standard deviation of ±20% and parameter range of 2.5 times the S.D. and validated with outcome data
RNS_ROS_ATP_inhib_Vmax	RNS/ROS ATP inhibition Vmax	Parameter range derived from (24)
RNS_ROS_cl_Vmax	Liver RNS/ROS baseline clearance Vmax	Assumed standard deviation of ±20% and parameter range of 2.5 times the S.D. and validated with outcome data
Basal_Stdzd_MitoETC_Flux	Basal value of mito ETC flux	Parameter range from healthy volunteer data (25)
Resp_Reserve_Scalar	Scaling coefficient representing reserve mitochondria function	Parameter range from healthy volunteer data (25)
CAS_apop_scale	Caspase-mediated apoptosis scaling constant	Parameter range derived from (26)
BA_uptake_Vmax	Bulk bile acid uptake Vmax	All transporters were assumed to have the same distribution as human BSEP reported in (27); similar expression ranges are also reported in (28); all uptake Vmax values are covariant
BA_baso_Vmax	Bulk bile acid basolateral transport Vmax	All transporters were assumed to have the same distribution as human BSEP reported in (27); similar expression ranges are also reported in (28); all basolateral Vmax values are covariant
BA_canal_Vmax	Bulk bile acid canalicular transport Vmax	All transporters were assumed to have the same distribution as human BSEP reported in (27); similar expression ranges are also reported in (28); all canalicular Vmax values are covariant
LCA_uptake_Vmax	LCA uptake Vmax	All transporters were assumed to have the same distribution as human BSEP reported in (27); similar expression ranges are also reported in (28); all uptake Vmax values are covariant
LCA_baso_Vmax	LCA basolateral transport Vmax	All transporters were assumed to have the same distribution as human BSEP reported in (27); similar expression ranges are also reported in (28); all basolateral Vmax values are covariant
LCA_canal_Vmax	LCA canalicular transport Vmax	All transporters were assumed to have the same distribution as human BSEP reported in (27); similar expression ranges are also reported in (28); all canalicular Vmax values are covariant
LCAamide_uptake_Vmax	LCA-amide uptake Vmax	All transporters were assumed to have the same distribution as human BSEP reported in (27); similar expression ranges are also reported in (28); all uptake Vmax values are covariant
LCAamide_baso_Vmax	LCA-amide basolateral transport Vmax	All transporters were assumed to have the same distribution as human BSEP reported in (27); similar expression ranges are also reported in (28); all basolateral Vmax values are covariant
LCAamide_canal_Vmax	LCA-amide canalicular transport Vmax	All transporters were assumed to have the same distribution as human BSEP reported in (27); similar expression ranges are also reported in (28); all canalicular Vmax values are covariant
LCAsulfate_uptake_Vmax	LCA-sulfate uptake Vmax	All transporters were assumed to have the same distribution as human BSEP reported in (27); similar expression ranges are also reported in (28); all uptake Vmax values are covariant
LCAsulfate_baso_Vmax	LCA-sulfate basolateral transport Vmax	All transporters were assumed to have the same distribution as human BSEP reported in (27); similar expression ranges are also reported in (28); all basolateral Vmax values are covariant
LCAsulfate_canal_Vmax	LCA-sulfate canalicular transport Vmax	All transporters were assumed to have the same distribution as human BSEP reported in (27); similar expression ranges are also reported in (28); all canalicular Vmax values are covariant
CDCA_uptake_Vmax	CDCA uptake Vmax	All transporters were assumed to have the same distribution as human BSEP reported in (27); similar expression ranges are also reported in (28); all uptake Vmax values are covariant
CDCA_baso_Vmax	CDCA basolateral transport Vmax	All transporters were assumed to have the same distribution as human BSEP reported in (27); similar expression ranges are also reported in (28); all basolateral Vmax values are covariant
CDCA_canal_Vmax	CDCA canalicular transport Vmax	All transporters were assumed to have the same distribution as human BSEP reported in (27); similar expression ranges are also reported in (28); all canalicular Vmax values are covariant
CDCAamide_uptake_Vmax	CDCA-amide uptake Vmax	All transporters were assumed to have the same distribution as human BSEP reported in (27); similar expression ranges are also reported in (28); all uptake Vmax values are covariant
CDCAamide_baso_Vmax	CDCA-amide basolateral transport Vmax	All transporters were assumed to have the same distribution as human BSEP reported in (27); similar expression ranges are also reported in (28); all basolateral Vmax values are covariant
CDCAamide_canal_Vmax	CDCA-amide canalicular transport Vmax	All transporters were assumed to have the same distribution as human BSEP reported in (27); similar expression ranges are also reported in (28); all canalicular Vmax values are covariant
CDCA_amidation_Vmax	CDCA amidation Vmax	Given same range as transporters due to lack of quantitative data
LCA_synthesis_Vmax	LCA synthesis Vmax	Assumed parameter range of ±2 orders of magnitude with ±50% standard deviation and validated with outcome data
LCAamide_sulfation_Vmax	LCA-amide sulfation Vmax	Given same range as transporters due to lack of quantitative data
canal_reg_scale	Canalicular transporter regulation exponent	Assumed parameter range of 0–8 with ±50% standard deviation and validated with outcome data
uptake_reg_scale	Uptake transporter regulation exponent	Assumed parameter range of 0–8 with ±50% standard deviation and validated with outcome data

## Results

### ***In Vitro*** Mitochondrial Toxicity Assay Results

**Fig. 2:**
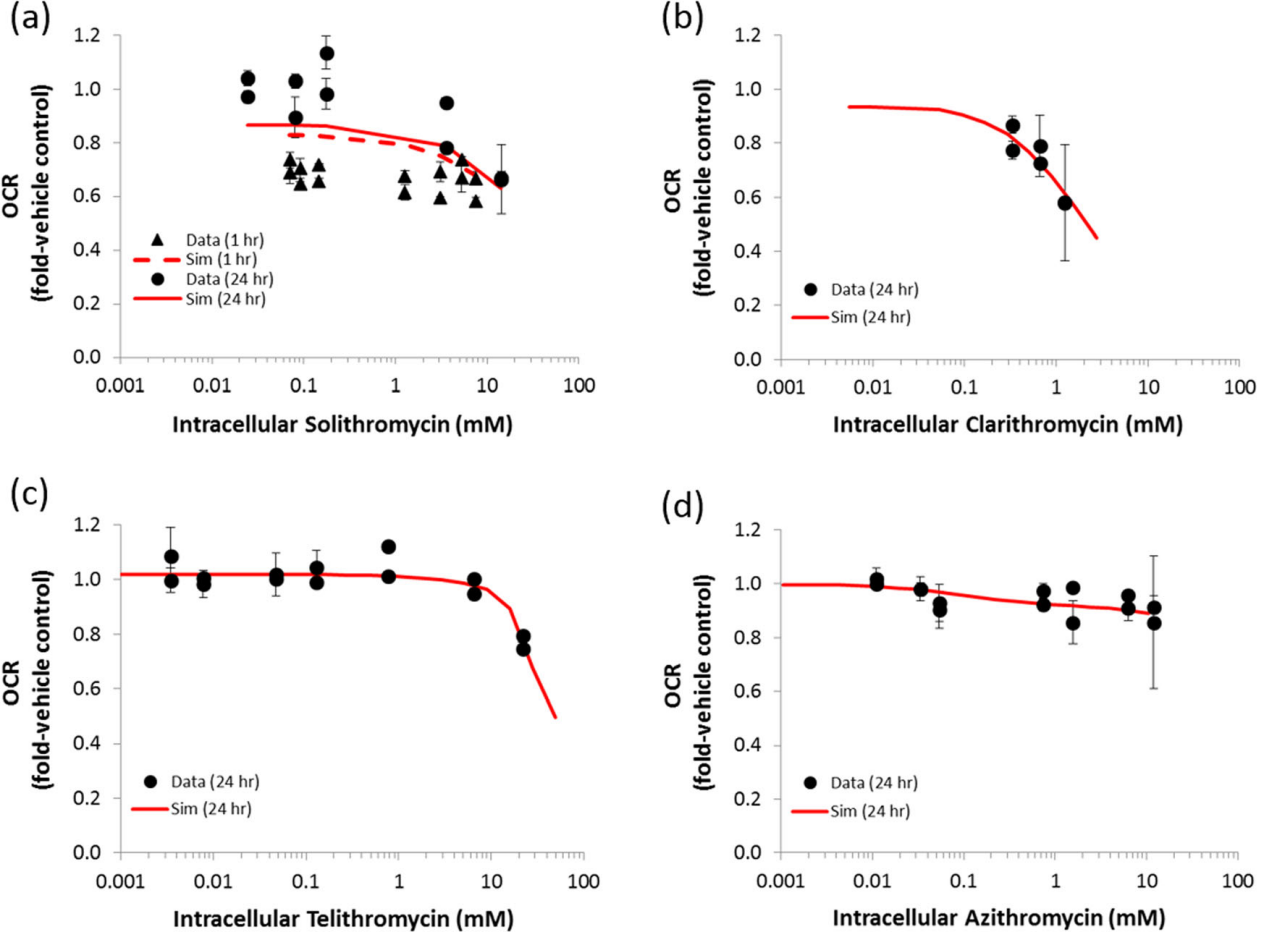
Comparison of simulation results in in vitro assay data to identify DILIsym parameter values that reproduce the concentration-dependent relationship between macrolides and mitochondrial toxicity. (**a**) solithromycin 1 and 24 h, (**b**) clarithromycin 24 h, (**c**) telithromycin 24 h, and (**d**) azithromycin 24 h. Symbols represent the measured oxygen consumption rate (OCR) in each independent experiment, and lines represent the simulated OCR.

In the mitochondrial respiration assay, solithromycin decreased basal oxygen consumption rate (OCR) in a concentration-dependent manner after 1 and 24 h incubation, whereas erythromycin did not inhibit cellular respiration at both time points. Telithromycin, clarithromycin, and azithromycin decreased basal OCR following 24 h incubation, but not after 1 h incubation (Fig. 2; 1 h data not shown). These data suggest that all the macrolides tested except for erythromycin are mitochondrial electron transport chain (ETC) inhibitors. Median ratios of intracellular concentration: media concentration measured by LC/MS/MS analysis were 263.4, 24.6, 4.3, 15.2, and 10.5 for solithromycin, erythromycin, telithromycin, clarithromycin, and azithromycin, respectively. DILIsym parameters for mitochondrial ETC inhibition for each compound were optimized to recapitulate intracellular concentrations *vs.* basal OCR data by simulating *in vitro*-like conditions within both MITOsym and DILIsym (Fig. 2). Reproduction of the OCR data defined solithromycin, clarithromycin, and azithromycin as ETC inhibitors with both a saturable ETC inhibition at low concentrations and complete ETC inhibition at higher concentrations. DILIsym parameters for complete inhibition (ETC inhibition 1) and saturable inhibition (ETC inhibition 3) were estimated simultaneously for these three compounds using DILIsym, whereas telithromycin’s effects on OCR were recapitulated with only the complete inhibition model using MITOsym (Table II).

**Table II:** Toxicity Parameters Utilized in the Simulation of the Five Macrolide Compounds in DILIsym v5A

Mechanism	Parameter	Unit	Value^f^				
			Solithromycin	Clarithromycin	Erythromycin	Telithromycin	Azithromycin
Mitochondrial dysfunction	Coefficient for ETC inhibition 1^a^	mol/mL	4 × 10^−5^	2.5 × 10^−6^	No inhibition	1.77 × 10^−4^	2.56 × 10^−4^
	Coefficient for ETC Inhibition 3^b^	mol/mL	1 × 10^−10^	1 × 10^−10^	No inhibition	No inhibition	5.00 × 10^−8^
	Max inhibitory effect for ETC inhibition 3^c^	dimensionless	0.35	0.3	No inhibition	No inhibition	0.35
Oxidative stress	RNS/ROS production rate constant 1^d^	mL/mol/hr	100,000	24,400	11,000	53,700	4000
Bile acid transporter inhibition	BSEP inhibition constant^e^	μM	28.2	59^g^	13^h^	5	No inhibition
	NTCP inhibition constant^e^	μM	No inhibition	No inhibition	No inhibition	No inhibition	No inhibition
	Inhibition constant for basolateral efflux^e^	μM	42.2	No inhibition^g^	No inhibition^h^	7.1^h^	No inhibition

^a^The inhibition constant for complete electron transport chain (ETC) inhibition

^b^The inhibition constant for partial ETC inhibition

^c^The maximal inhibitory effect for partial ETC inhibition

^d^The first order rate constant for the production of reactive nitrogen/oxygen species

^e^IC_50_ values; default assumption is mixed inhibition type with α = 5, based on the experience of the DSS team. For basolateral efflux, the more potent value between MRP3 and MRP4 were employed as a conservative approach

^f^Values shown in the table for DILIsym input parameters should not be interpreted in isolation with respect to clinical implications, but rather, should be combined with exposure in DILIsym® to produce simulations that have predictive and insightful value

^g^Vermeer 2016 (29); reports no MRP3 inhibition but does not report MRP4

^h^Morgan 2013 (30)

### ***In Vitro*** Oxidative Stress Assay Results

**Fig. 3:**
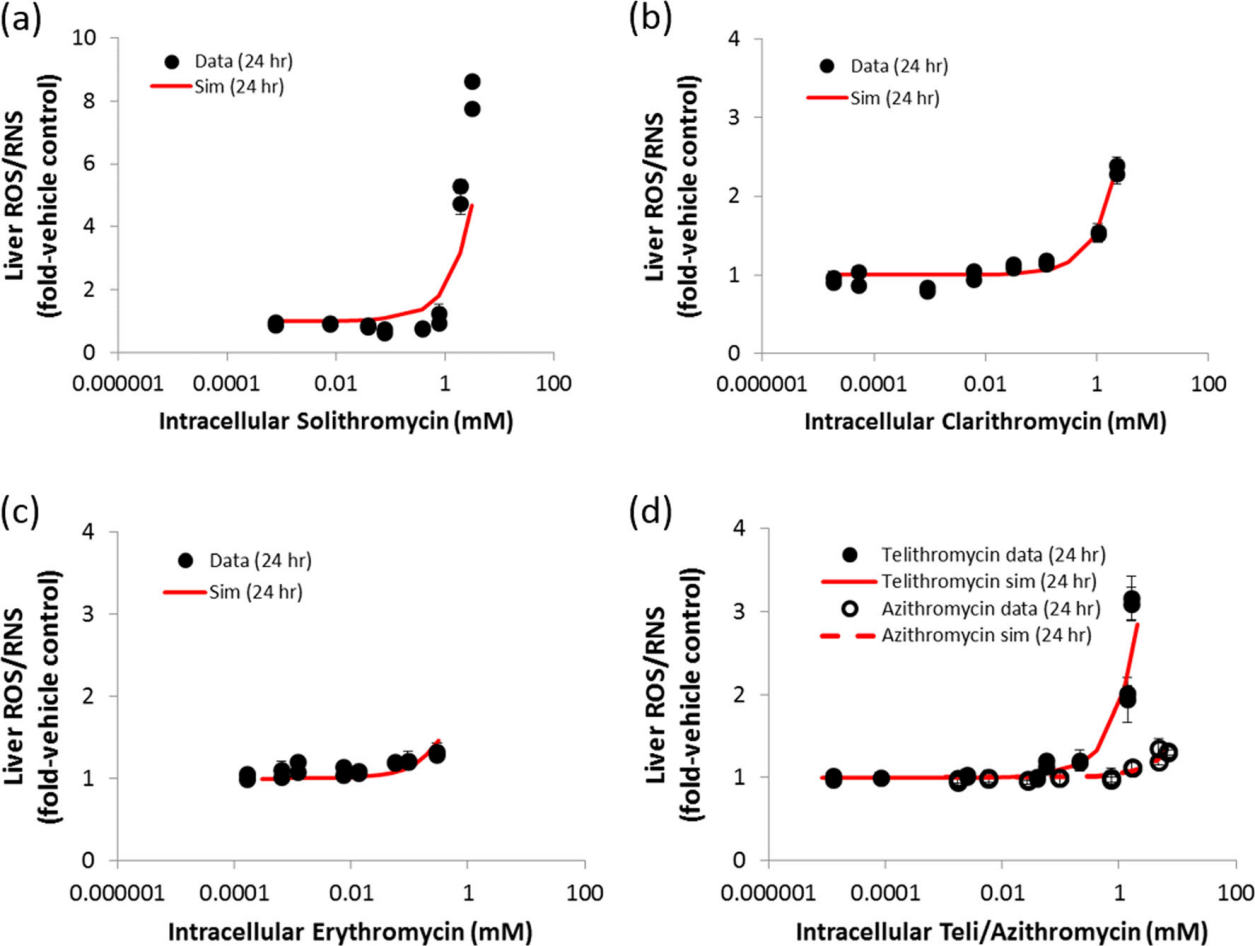
Comparison of simulation results and in vitro assay data to identify DILIsym parameter values that reproduce the concentration-dependent relationship between macrolides and oxidative stress (**a**) solithromycin 24 h, (**b**) clarithromycin 24 h, (**c**) erythromycin 24 h, and (**d**) telithromycin and azithromycin 24 h. Symbols represent the measured hepatic reactive oxygen/nitrogen species (ROS/RNS) in each independent experiment, and lines represent the simulated hepatic ROS/RNS.

All five macrolides increased RNS/ROS in a concentration-dependent manner after 24 h incubation, but not following 1 h incubation (Fig. 3; 1 h data not shown). These data suggest that the tested macrolides can elicit oxidative stress with varying potencies. Median ratios of intracellular concentration: media concentration measured by LC/MS/MS analysis were 78.5, 22.2, 4.34, 3.1, and 297 for solithromycin, erythromycin, telithromycin, clarithromycin, and azithromycin, respectively. DILIsym parameters for production of RNS/ROS were optimized to recapitulate intracellular concentrations *vs.* cellular RNS/ROS data by simulating *in vitro*-like conditions within DILIsym (Fig. 3, Table I).

### ***In Vitro*** Bile Acid Transporter Inhibition Assay Results

All five macrolides inhibited multiple bile acid transporters with varying potencies. Inhibition constants are presented in Table II.

### Simulation Results

The results for the v4A_1 SimPops simulations for each compound are shown in Table III, compared to the observed clinical frequency of ALT elevations. DILIsym accurately represented the observed frequency of ALT elevations for three of the five macrolide antibiotics. Solithromycin, erythromycin, and clarithromycin were all correctly predicted to cause low-frequency ALT elevations in the v4A_1 SimPops. The hepatocyte loss in these simulations was not sufficient to cause plasma bilirubin to increase above 2x the upper limit of normal; thus, no Hy’s Law cases were predicted for these drugs, consistent with clinical data. However, telithromycin and azithromycin were not predicted to cause ALT elevations >3 X ULN even though some clinical hepatotoxicity has been observed for both of these compounds.

**Table III:** Results in the v4A_1 SimPops for Each of the Five Macrolides in DILIsym v5A Compared to Reported Clinical data. Observed Data are from the Literature (3,10,31)

Compound	Protocol	Peak ALT >3X ULN	
		Observed	Simulated^**^
Solithromycin	Oral (CE01–300)	5.4%^a^	3.9%
		(22/411)	(11/285)
	IV-to-Oral (CE01–301)	9.1%^b^	6.0%
		(38/417)	(17/285)
Clarithromycin	500 mg BID 7 days	1–2%	2.8%
			(8/285)
Erythromycin	500 mg	1–2%	2.8%
	QID 10 days		
			(8/285)
Telithromycin	800 mg QD 10 days	~0.5%	0%
Azithromycin	500 mg QD day 1	1.2%	0%
	250 mg QD days 2–5		

Upper limit of normal (ULN) in DILIsym is 40 U/L

^a^(9); 2.8% among patients with normal baseline ALT

^b^(8); 6.6% among patients with normal baseline ALT

Results for the Mechanistic Investigation Simulations are shown in Table IV. For solithromycin and clarithromycin, the largest decline in simulated ALT elevations was observed when the ETC inhibition effect was omitted, suggesting that this mechanism was the most important in explaining the observed ALT elevations for these two compounds. By contrast, for erythromycin, the elimination of bile acid transporter inhibition effects led to the largest decline in simulated ALT elevation frequency, suggesting that this mechanism best explains the observed erythromycin ALT elevations. For telithromycin and azithromycin, mechanistic investigation simulations were not run since no ALT elevations greater than 3-fold above the ULN occurred in the v4A_1 SimPops simulations. The likely mechanisms for the five macrolide antibiotics, as predicted by the *in vitro* assays and the Mechanistic Investigation simulations, are shown in Table V.

**Table IV:** Mechanistic Investigation Simulations for Each of the Three Macrolides for Which ALT Elevations were Predicted by DILIsym

Compound	Mechanism(s) On	Mechanism Off	Simulated	
			ALT >3X ULN^a^	
			Oral	IV-to-Oral
Solithromycin	All	–	11/285	17/285
	(ETCi, ROS, BAi)			
	ETCi, ROS	BAi	6/285	8/285
	BAi, ROS	ETCi	0/285	0/285
	BAi, ETCi	ROS	11/285	17/285
Erythromycin	All	–	8/285	N/A
	(ROS, BAi)			
	ROS	BAi	1/285	N/A
	BAi	ROS	7/285	N/A
Clarithromycin	All	–	8/285	N/A
	(ETCi, ROS, BAi)			
	ETCi, ROS	BAi	3/285	N/A
	BAi, ROS	ETCi	0/285	N/A
	BAi, ETCi	ROS	8/285	N/A

For erythromycin, ETC inhibition was not used as a mechanism; this is why there is no simulation with ETCi off

^a^The upper limit of normal (ULN) of ALT in DILIsym is 40 U/L. Every individual in DILIsym begins the simulation at 30 U/L plasma ALT

**Table V:** Most Likely Mechanism of Toxicity Suggested by the Simulation Results for Each Macrolide Antibiotic

DILI mechanism	Solithromycin	Clarithromycin	Erythromycin	Telithromycin	Azithromycin
Mitochondrial dysfunction	**Predominant**	**Predominant**	None	None	Plausible
Oxidative stress	None	None	Minor	None	None
Bile acid transporter inhibition	Minor	Minor	**Predominant**	Plausible	None
Mechanism not included in DILIsym	Unlikely	Unlikely	Unlikely	**Plausible**	**Plausible**

The mechanism suggested by DILIsym as the most likely to contribute to the observed toxicity is rendered in bold

## Discussion

Macrolide antibiotics have been associated with varying levels of liver injury, but the underlying mechanisms have not been elucidated. In the current study, QST modeling was employed to integrate *in vitro* mechanistic toxicity data, *in vivo* drug exposure, and underlying biochemistry. Using this approach, DILIsym correctly predicted the frequency of ALT elevations for three of the five macrolides: clarithromycin, solithromycin, and erythromycin. Interestingly, for the three macrolides, different mechanisms were implicated in the observed toxicity; simulations suggest that solithromycin and clarithromycin toxicity is caused mainly by mitochondrial ETC inhibition while erythromycin toxicity is caused predominantly by bile acid accumulation. Furthermore, the fact that telithromycin and azithromycin toxicity was not predicted by DILIsym suggests the presence of a different mechanism that is not represented in DILIsym, though bile acid accumulation did lead to some predictions of sub-clinical ALT elevations for telithromycin (data not shown). This is an interesting result because these five molecules are all in the same class of drug and all somewhat structurally similar; however, they are clearly mechanistically distinct from one another with regard to their hepatic effects. The simulation results therefore demonstrate that one should not draw conclusions about the mechanisms of toxicity – or about the frequency thereof – for a molecule based on the fact that it is part of the same class of drug as another molecule that causes toxicity via a known mechanism.

For telithromycin, in particular, it was not surprising that DILIsym failed to predict ALT elevations. ALT elevations were rather rare in the clinic with telithromycin, and indeed ALT elevations were less frequent during treatment with telithromycin than with the other macrolides (31). The severe toxicity observed with telithromycin was a very rare event (i.e., 1 in 20,000) that may not be able to be predicted in a 285-individual simulated population. There are potentially effects caused by metabolites that could have been missed in this analysis; furthermore, if the PBPK simulation underestimated liver partitioning this could also explain an underprediction. As previously mentioned, it is also possible that the cellular stress that occurs with telithromycin may be caused by a mechanism that is not included in DILIsym currently. This is almost certainly the case with azithromycin; while widely considered to be the safest of the macrolide antibiotics (4,32,33), azithromycin has been reported to cause ALT elevations in 1–2% of the population. However, these elevations often occur after the cessation of dosing (34,35), a phenomenon that cannot be explained by either bile acid accumulation, interference with mitochondrial respiration, or generation of oxidative stress, all of which require drug to be present in order for toxicity to develop. More research should be done to propose a plausible hepatotoxicity mechanism that causes a latent effect that manifests after the cessation of treatment before such a model can be assessed with DILIsym. In the case of telithromycin, inhibition of the “inflammatory reflex” has been proposed as a plausible mechanism for its toxicity based upon its inhibition of nicotinic acetylcholine receptors by the pyridine moiety contained in the side chain of telithromycin (36,37). More recently, the steps following the release of acetylcholine, activation of the nACh receptors, and hepatocyte regeneration has been described (38). Blocking of nACh receptor activation by telithromycin would block protective hepatocyte regeneration.

One of the more interesting aspects of this work is how the *in vitro* assays did not map directly to the mechanisms that were responsible for toxicity. For example, each of the five macrolides demonstrated some response in the oxidative stress assay, but ROS was found to contribute only to erythromycin toxicity by the simulations. The combination of *in vitro* assay results with measures of exposure has been shown to produce an increased ability to predict liver toxicity beyond that of the assay alone (30); the combination of *in vitro* assay results, exposure estimates, and known biological variability contained by QST models such as DILIsym can provide considerably greater mechanistic insight than the assays results alone.

In patients treated with solithromycin, increased ALT was normalized with continued dosing or soon after the end of treatment (8). These data suggest that the liver was able to adapt to the mild liver injury instigated by solithromycin. One proposed mechanism for adaptation in liver injury is mitochondrial biogenesis; this is a key mechanism for recovery from mitochondrial stress in muscles resulting from exercise (39) and has been observed in mouse hepatocytes after exposure to the ETC inhibitor rotenone (40) and in rat liver after dosing with the DILI-inducing drug valproate (41,42). Solithromycin and clarithromycin, as drugs whose ALT elevations are largely driven by mitochondrial effects, would be more likely to respond with this adaptive mechanism, which may be less relevant to the other macrolides. DILIsym does not yet include mitogenesis and several other potentially important adaptive processes that may mitigate some of the toxic response to drugs. Future simulation work will incorporate mitochondrial biogenesis into DILIsym and compare the effects of this adaptive mechanism on the simulation results.

One limitation of this work is that the metabolites of the macrolides were not investigated for their potential toxic effects. The results suggest that it is unlikely that metabolites of solithromycin, clarithromycin, and erythromycin are contributing to the observed ALT elevations; the liver responses for these molecules were adequately explained by the effects of the parent compound. However, metabolite effects may be contributing to azithromycin and telithromycin toxicity, since these compounds’ toxic responses were not adequately explained by parent effects due to the mechanisms represented in DILIsym. Furthermore, the simulations were conducted in a SimPops intended to represent normal healthy volunteers; the ALT elevations were almost all observed in individuals with some sort of bacterial infection. It is unclear whether infected individuals demonstrate a different level of drug exposure for many of the macrolides, though elderly individuals and individuals with community-acquired bacterial pneumonia have been shown to have a higher plasma concentration of telithromycin than normal healthy volunteers (43). Differences in exposure between healthy volunteers and infected individuals may help explain some of the observed telithromycin and azithromycin toxicity; a better understanding of the differences in exposure between infected and healthy individuals is necessary. Uncertainty in the *in vitro-in vivo* extrapolation (IVIVE) process for the toxicity parameters is also a plausible reason for the lack of predictivity for telithromycin and azithromycin; if the estimate of intracellular:extracellular concentration ratio derived from the mass spectrometry assay is significantly different from that which occurs *in vivo,* for example, the toxicity parameter derivation may be affected as a result. However, the magnitude of the assay uncertainty necessary for this effect to be able to explain the missed predictions of telithromycin and azithromycin is quite large, as suggested by dose escalation simulations conducted on both compounds (data not shown). As a result, we view this as a less likely contributor than alternative mechanisms, patient effects, or metabolite effects.

## Conclusions

In conclusion, DILIsym was used to contextualize novel *in vitro* experiments and assess the likelihood that five macrolide antibiotics cause serum ALT elevations by oxidative stress, mitochondrial toxicity, and bile acid accumulation. DILIsym found that solithromycin and clarithromycin ALT elevations can be primarily accounted for by mitochondrial ETC inhibition whereas erythromycin ALT elevations can be primarily accounted for by inhibition of bile acid transporters. Bile acid transporter inhibition may also in part account for ALT elevations caused by telithromycin, but the model predictions were poor for both telithromycin and azithromycin. This may reflect effects of metabolites of these drugs or involvement of mechanisms not included in DILIsym. This research demonstrates that despite the fact that these five drugs are all in the same class, they are mechanistically distinct from one another concerning their hepatic adverse effects.

## Electronic supplementary material

ESM 1 (MAT 82 kb)

ESM 2 (MAT 82 kb)

ESM 3 (MAT 82 kb)

ESM 4 (MAT 3384 kb)

ESM 5 (MAT 83 kb)

ESM 6 (DOCX 684 kb)
