# Supplementary material for: Analyzing the Mechanisms Behind Macrolide Antibiotic-Induced Liver Injury Using Quantitative Systems Toxicology Modeling
Source: Pharm Res. 2019 Feb 7;36(3):48. doi: 10.1007/s11095-019-2582-y (PMC6373306; doi:10.1007/s11095-019-2582-y)
Supplement: Supplementary file 6 — (DOCX 684 kb) [file 11095_2019_2582_MOESM6_ESM.docx]

**Supplement A** – PBPK Modeling Methods

All compound PBPK models were optimized within the PBPK sub-model of DILIsym v5A. The parameter set that best fit the data was determined by genetic algorithm-based global optimization with small subsequent manual changes; the model fits shown represent the global minimum of a least-squares fit to the plasma PK data as calculated by the genetic algorithm, in some cases with minor modifications made in order to fit other data as described below. The parameters listed in Tables A1, A3, A5, A7, and A9 were fitted simultaneously to the plasma PK data shown here for each compound. The portion of the process specific to each drug is listed below.

*Solithromycin*

The current PBPK model was constructed to represent solithromycin doses ≥ 400 mg; complex nonlinear pharmacokinetic behaviors were observed at the lower dose levels of solithromycin, but such lower doses were not employed in the phase 3 clinical trials and thus excluded from the current modeling. Solithromycin tissue distribution was represented by partition coefficients (first-order, gradient driven distribution), which were estimated based on its physicochemical properties. Active transport was assumed to contribute minimally to the liver distribution of solithromycin because in vitro data showed that solithromycin is not transported by OATP1B1, OATP1B3, NTCP, and OCT. Metabolism is the major elimination route of solithromycin. Because solithromycin PK profiles were linear at targeted dose ranges (≥ 400 mg), hepatic metabolism was assumed to be linear. Therefore, the metabolic K_m_ was set to a very high value (1000 µM). Metabolic V_max_ was optimized because *in vitro* metabolism data could not recapitulate plasma PK profiles after IV and oral doses. PK data from repeat dose studies showed that plasma solithromycin exposure was increased after multiple doses compared to that after a single dose; *in vitro* studies demonstrated that solithromycin can inhibit its own metabolism by time-dependent inhibition of CYP3A4. In the current PBPK representation, solithromycin metabolism was represented by two metabolic pathways; Metabolite A represents the CYP3A4-mediated metabolism pathway which is subject to time-dependent inhibition. Metabolite B represents lumped pathways of non-CYP3A4 mediated metabolism which are not subject to time-dependent inhibition. The relative contribution of the CYP3A4-mediated pathway (Metabolite A) and the non-CYP3A4-mediated pathway (Metabolite B) to overall metabolism were determined to be 4:1 based on the sensitivity analysis. Parameters governing time-dependent inhibition were optimized to repeat dose clinical PK data. Metabolites were assumed not to contribute to liver toxicity, and thus were not tracked. *In vivo* mass balance data indicated that solithromycin is also excreted into urine unchanged, but does not undergo biliary excretion. Renal clearance value was obtained from clinical data, whereas biliary excretion of the parent was assumed to be negligible. Solithromycin has a measured plasma fraction unbound in human of 19% and a blood:plasma ratio of 0.76. Because the measured plasma fraction unbound could not recapitulate plasma PK profiles after IV infusion, an adjusted plasma fraction unbound value was employed (Poulin et al., 2016). Parameters involved in intestinal absorption were also optimized.

Optimization was conducted until the plasma time courses of solithromycin were in agreement with the solithromycin plasma concentration-time data after IV infusion (400 mg) and multiple oral dosing (400 mg QD). Following optimization, model-simulated plasma AUC and plasma C_max_ were evaluated to ensure consistency with observed data. The solithromycin PBPK model was validated against the plasma concentration-time data from the Oral and the IV-to-Oral protocols. The full list of parameters, including those optimized and those derived from experimental data, are listed in Table A1. The optimization and validation pots are shown in Figures A1 and A2, respectively; a comparison of PK parameters between the simulation and the literature is shown in Table A2. Overall, the DILIsym PBPK sub-model simulation showed a good fit to the optimization data. The simulated liver-to-plasma C_max_ ratio (K_p_) values of 2.4 (IV infusion) and 7.7 – 9.6 (PO) were similar to a K_p_ value of 7.3, which was predicted from the physicochemical properties of solithromycin.

*Erythromycin*

For erythromycin, *in vitro* metabolism data were available from the literature (RILEY); these data were used to determine a V_max_ and K_m_ for erythromycin metabolism. Some tissue partitioning data were available from the literature (Krasniqi et al., 2012); these data were used to parameterize the muscle tissue partition coefficient, while others were optimized. Gut absorption parameters and blood:plasma ratio were available from the literature as well (Mather et al., 1981). Liver uptake Vmax and permeability, other partition coefficients, and biliary and renal clearance parameters were optimized to the plasma data. Following optimization, model-simulated plasma AUC and plasma C_max_ were calculated and compared to measured clinical data to ensure consistency with observed data. The simulated liver:plasma concentration ratio was compared to values from the literature, which estimate the erythromycin liver:plasma concentration ratio in rats as between 5 and 20 (Hanada et al., 1997; Kohno et al., 1989). Comparison to the calculated intracellular:extracellular ratio in HepG2 cells was also used to validate the PBPK representation. The full list of parameters, including those optimized and derived from experimental data, are listed in Table A3.

The plasma time course used to optimize the erythromycin PBPK model was a 500 mg QID study obtained from the literature (Krasniqi et al., 2012). The result of this optimization is shown in Figure A3; a comparison of PK parameters between the simulation and the literature is shown in Table A4.

*Telithromycin*

The telithromycin representation in DILIsym was based on a previously published PBPK model for telithromycin (Vieira et al., 2012). The relative contribution of the CYP3A4-mediated pathway (Metabolite A) and the non-CYP3A4-mediated pathway (Metabolite B) to overall metabolism was assumed to be 1:1 based on previously reported information ("Telithromycin"), though the Vmax values for these metabolites were optimized to plasma data. Parameters governing inhibition of metabolism to Metabolite A were optimized to repeat dose PK data. Renal clearance was optimized within a range of reported values from literature, as was biliary clearance and plasma fraction unbound. Parameters involved in intestinal absorption were also optimized, as were parameters governing metabolite distribution and excretion. Optimization was conducted until the plasma time courses of telithromycin and its metabolite were in agreement with the observed data after 800 mg daily oral dosing; the excretion balance of telithromycin was also used in the optimization scheme. The full list of parameters, including those optimized and those derived from experimental data, are listed in Table A5.

The plasma data used to optimize the telithromycin model was from Namour et al. (Namour et al., 2001); the data included a 7-day plasma time course for both telithromycin and its main metabolite Ro-76363 with 400, 800, and 1600 mg QD dosing. The model results can be seen in Figure A4; Table A6 contains the comparison between simulated and observed PK parameters.

*Clarithromycin*

For clarithromycin, tissue partition coefficients for gut, liver, muscle, and other tissue were optimized because those estimated using physicochemical properties (Rodgers and Rowland, 2006; Rodgers et al., 2005) did not recapitulate clarithromycin disposition after IV infusion. A scaling factor that modulates all the initial tissue partition coefficients by the same fold was optimized to clinical PK data after IV and PO administration. Metabolic V_max_ was optimized because *in vitro* metabolism data could not recapitulate plasma PK profiles after IV and oral doses. Parameters governing inhibition of metabolism to Metabolite A were also optimized to repeat dose PK data. Renal clearance was optimized to recapitulate clinically observed urinary recovery of clarithromycin (Chu et al., 1992a), whereas biliary excretion of the parent was assumed to be negligible. Parameters involved in intestinal absorption were also optimized. Optimization was conducted until the plasma time courses of clarithromycin were in agreement with the observed data after IV infusion (250 mg) and oral doses (250 mg and 500 mg). Following optimization, model-simulated plasma AUC and plasma C_max_ were evaluated to ensure consistency with observed data. The full list of parameters, including those optimized and those derived from experimental data, are listed in Table A7.

Simulation results from the DILIsym PBPK representation of clarithromycin compared to plasma data are shown in Figure A5, while the comparison to measured PK parameters is shown in Table A8. The DILIsym PBPK sub-model simulation showed a good fit to the clinical data (Figure A4, Table A8). In simulations of oral clarithromycin protocols, 27 – 28 % of dose was excreted into urine unchanged, consistent with clinically observed urinary recovery of 20 – 30 %. The simulated liver-to-plasma C_max_ ratio (K_p_) values were of 4.8 (IV infusion) and 15 – 15.2 (PO).

*Azithromycin*

Literature suggests that azithromycin partitions strongly into the liver while weakly partitioning into other tissues (Matzneller et al., 2013); a partition coefficient for muscle was based on the reported value while other tissue:blood partition coefficients have been optimized. Azithromycin metabolism was represented by a single lumped pathway. Renal and biliary clearance parameters were likewise optimized. Parameters used for the azithromycin model are presented in Table A9.

Figure A6 shows the results of the PBPK model compared to 500 mg QD plasma data (Matzneller et al., 2013); the comparison between simulated and observed PK parameters is shown in Table A10. The PBPK model is a good fit to the published data.

**References**

Chu, S., Wilson, D.S., Deaton, R.L., Mackenthun, A.V., Eason, C.N., and Cavanaugh, J.H. (1993). Single- and multiple-dose pharmacokinetics of clarithromycin, a new macrolide antimicrobial. J. Clin. Pharmacol. *33*, 719–726.

Chu, S.Y., Sennello, L.T., Bunnell, S.T., Varga, L.L., Wilson, D.S., and Sonders, R.C. (1992a). Pharmacokinetics of clarithromycin, a new macrolide, after single ascending oral doses. Antimicrob. Agents Chemother. *36*, 2447–2453.

Chu, S.Y., Deaton, R., and Cavanaugh, J. (1992b). Absolute bioavailability of clarithromycin after oral administration in humans. Antimicrob. Agents Chemother. *36*, 1147–1150.

Davey, P.G. (1991). The pharmacokinetics of clarithromycin and its 14-OH metabolite. J. Hosp. Infect. *19 Suppl A*, 29–37.

Hanada, E., Ohtani, H., Kotaki, H., Sawada, Y., and Iga, T. (1997). Determination of erythromycin concentrations in rat plasma and liver by high-performance liquid chromatography with amperometric detection. J. Chromatogr. B. Biomed. Sci. App. *692*, 478–482.

Kohno, Y., Yoshida, H., Suwa, T., and Suga, T. (1989). Comparative pharmacokinetics of clarithromycin (TE-031), a new macrolide antibiotic, and erythromycin in rats. Antimicrob. Agents Chemother. *33*, 751–756.

Krasniqi, S., Matzneller, P., Kinzig, M., Sörgel, F., Hüttner, S., Lackner, E., Müller, M., and Zeitlinger, M. (2012). Blood, tissue, and intracellular concentrations of erythromycin and its metabolite anhydroerythromycin during and after therapy. Antimicrob. Agents Chemother. *56*, 1059–1064.

Lancaster, C.S., Bruun, G.H., Peer, C.J., Mikkelsen, T.S., Corydon, T.J., Gibson, A.A., Hu, S., Orwick, S.J., Mathijssen, R.H.J., Figg, W.D., et al. (2012). OATP1B1 polymorphism as a determinant of erythromycin disposition. Clin. Pharmacol. Ther. *92*, 642–650.

Mather, L.E., Austin, K.L., Philpot, C.R., and McDonald, P.J. (1981). Absorption and bioavailability of oral erythromycin. Br. J. Clin. Pharmacol. *12*, 131–140.

Matzneller, P., Krasniqi, S., Kinzig, M., Sörgel, F., Hüttner, S., Lackner, E., Müller, M., and Zeitlinger, M. (2013). Blood, tissue, and intracellular concentrations of azithromycin during and after end of therapy. Antimicrob. Agents Chemother. *57*, 1736–1742.

Namour, F., Wessels, D.H., Pascual, M.H., Reynolds, D., Sultan, E., and Lenfant, B. (2001). Pharmacokinetics of the new ketolide telithromycin (HMR 3647) administered in ascending single and multiple doses. Antimicrob. Agents Chemother. *45*, 170–175.

Poulin, P., Burczynski, F.J., and Haddad, S. (2016). The Role of Extracellular Binding Proteins in the Cellular Uptake of Drugs: Impact on Quantitative In Vitro-to-In Vivo Extrapolations of Toxicity and Efficacy in Physiologically Based Pharmacokinetic-Pharmacodynamic Research. J. Pharm. Sci. *105*, 497–508.

Quinney, S.K., Zhang, X., Lucksiri, A., Gorski, J.C., Li, L., and Hall, S.D. (2010). Physiologically based pharmacokinetic model of mechanism-based inhibition of CYP3A by clarithromycin. Drug Metab. Dispos. Biol. Fate Chem. *38*, 241–248.

Riley, R.J., and Howbrook, D. (1997). In vitro analysis of the activity of the major human hepatic CYP enzyme (CYP3A4) using [N-methyl-14C]-erythromycin. J. Pharmacol. Toxicol. Methods *38*, 189–193.

Rodgers, T., and Rowland, M. (2006). Physiologically based pharmacokinetic modelling 2: predicting the tissue distribution of acids, very weak bases, neutrals and zwitterions. J. Pharm. Sci. *95*, 1238–1257.

Rodgers, T., Leahy, D., and Rowland, M. (2005). Physiologically based pharmacokinetic modeling 1: predicting the tissue distribution of moderate-to-strong bases. J. Pharm. Sci. *94*, 1259–1276.

Vieira, M.L.T., Zhao, P., Berglund, E.G., Reynolds, K.S., Zhang, L., Lesko, L.J., and Huang, S.-M. (2012). Predicting drug interaction potential with a physiologically based pharmacokinetic model: a case study of telithromycin, a time-dependent CYP3A inhibitor. Clin. Pharmacol. Ther. *91*, 700–708.

Telithromycin.

Figure A1. Simulated and observed plasma concentrations of solithromycin after a single IV dose at 400 mg (30-min infusion) (a), a single oral dose at 400 mg (b), and multiple oral doses at 400 mg (day 7) (c). Black circles represent the mean data, which were used for the optimization of the PBPK sub-model. Red lines represent simulated solithromycin concentrations.


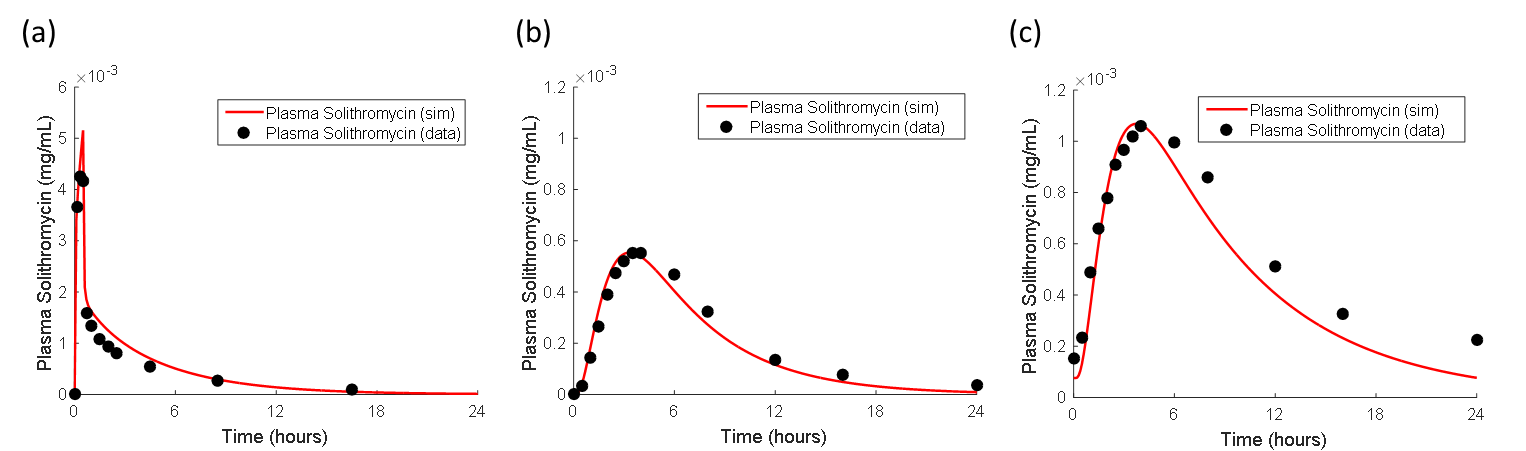


Figure A2. Simulated and observed plasma concentrations of solithromycin with the IV-to-Oral protocol (a), and the Oral protocol (b). Black circles represent the mean data, which were not used for the optimization of the PBPK sub-model (validation dataset). Red lines represent simulated solithromycin concentrations.


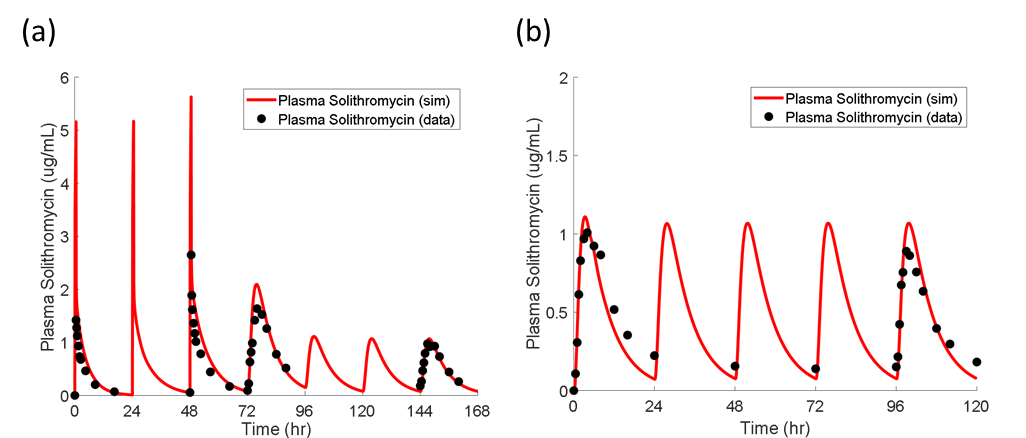


Figure A3. Comparison between simulated and observed (Krasniqi et al., 2012) plasma time course after 3 days of 500 mg QID erythromycin dosing.


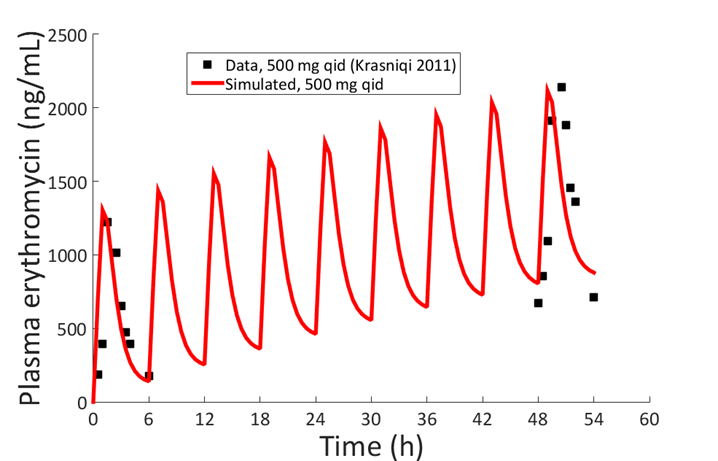


Figure A4. Simulated and observed plasma concentrations of a) telithromycin and b) the main telithromycin metabolite Ro-76363 after 10 days of oral 800 mg QD dosing. The data are from Namour et al. (Namour et al., 2001)

**
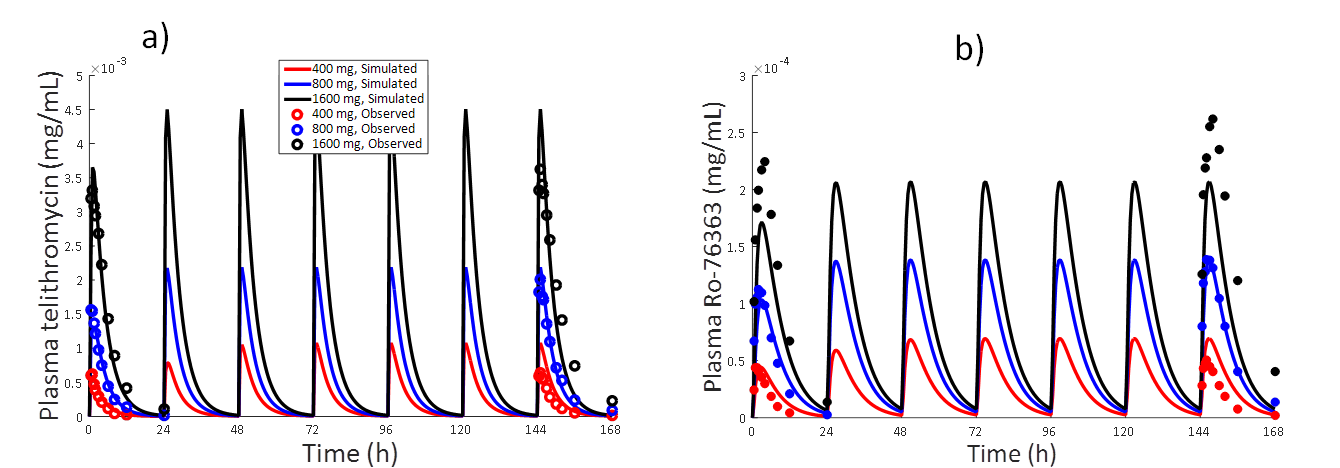
**

Figure A5. Simulated and observed plasma concentrations of clarithromycin after a single IV dose at 250 mg (45-min infusion) (a), a single oral dose at 250 and 500 mg (b), or multiple oral doses at 250 and 500 mg (day 4; 7 doses of clarithromycin were given q 12hr) (c). The data (Chu et al., 1993, 1992a) represent the mean.


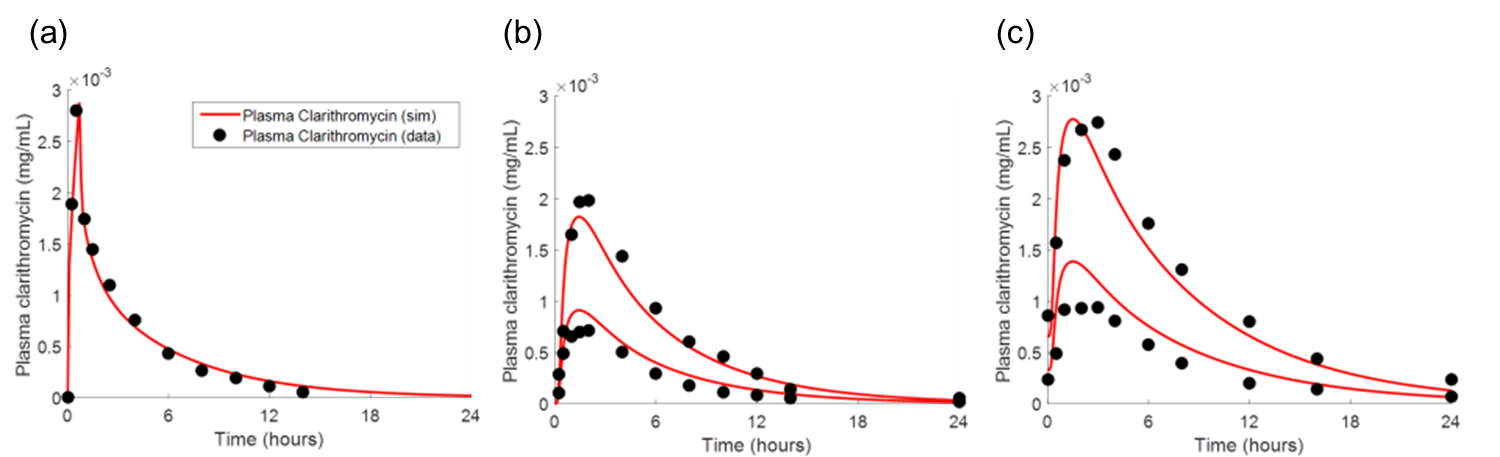


Figure A6. Comparison between simulation and observation of plasma time course after a 500 mg QD dose of azithromycin for 7 days.


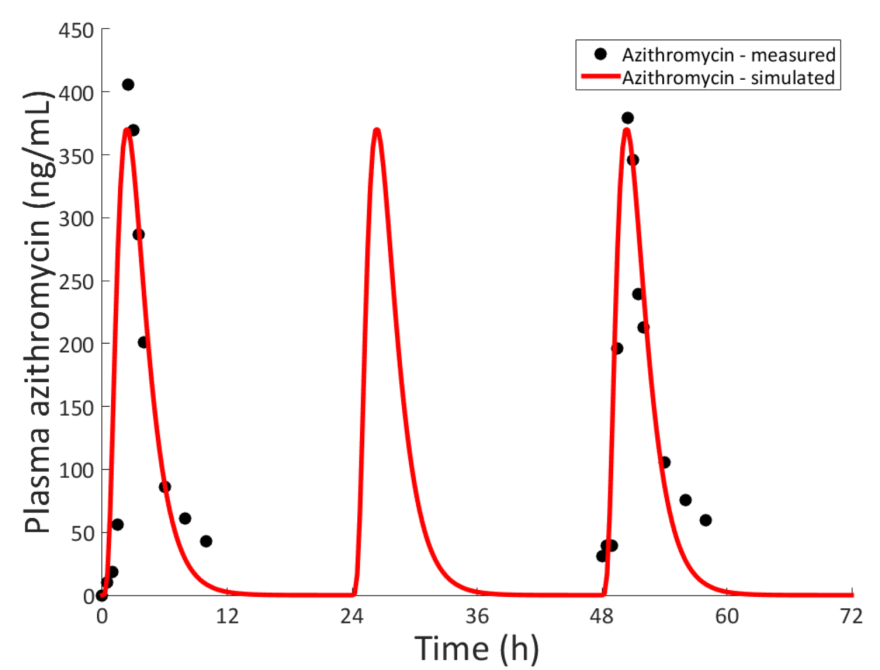


Table A1. Parameters used in the DILIsym PBPK sub-model for solithromycin.

| Parameter | Unit | Value | Source |
| --- | --- | --- | --- |
| Compound W absorption from gut | 1/hour | 0.94 | Optimization |
| Compound W dissolution | 1/hour | 0.82 | Optimization |
| Compound W gastric emptying | 1/hour | 1.6 | Optimization |
| Compound W rate of elimination in feces | 1/hour | 0.1 | Optimization |
| Compound W molecular weight | g/mol | 845.01 | Measured |
| Compound W blood to plasma | dimensionless | 0.76 | Measured |
| Compound W fraction unbound plasma | dimensionless | 0.919 | Calculated (Poulin 2016) |
| Compound W gut to blood | dimensionless | 4.31 | Rodgers Eq (Rodgers and Rowland, 2006; Rodgers et al., 2005) |
| Compound W liver to blood | dimensionless | 5.54 | Rodgers Eq (Rodgers and Rowland, 2006; Rodgers et al., 2005) |
| Compound W muscle to blood | dimensionless | 2.35 | Rodgers Eq (Rodgers and Rowland, 2006; Rodgers et al., 2005) |
| Compound W other tissue to blood | dimensionless | 3.49 | Rodgers Eq (Rodgers and Rowland, 2006; Rodgers et al., 2005) |
| Compound W renal clearance | mL/hour/kg^0.75 | 293.5 | Clinical data |
| Compound W biliary excretion Vmax | mg/hour/kg^0.75 | 0 | Cempra report |
| Compound W biliary excretion Km | mg/mL | 1.00E+10 | Cempra report |
| Km(Compound W metabolite A) | mol/mL | 1.00E-06 | Assumed (linear) |
| Vmax(Compound W metabolite A) | mol/hour/kg^0.75 | 0.002 | Optimization |
| Compound W delay time constant  (metabolite A induction) | 1/hour | 0.0004 | Optimization |
| Compound W metabolite A induction Vmax | 1/hour | -0.75 | Optimization |
| Compound W metabolite A induction Km | mg/mL | 1.6E-05 | Optimization |
| Compound W metabolite A induction Hill | dimensionless | 30 | Optimization |
| Km(Compound W metabolite B) | mol/mL | 1.00E-06 | Assumed (linear) |
| Vmax(Compound W metabolite B) | mol/hour/kg^0.75 | 0.00049 | Optimization |

Table A2. Comparison between observed and simulated PK parameters for solithromycin.

| Protocol | Day | Dose | C_max_ | | | AUC | | |
| --- | --- | --- | --- | --- | --- | --- | --- | --- |
|  |  |  | **Observed**  **(µM)** | **Simulated (µM)** | **Sim/Obs**  **(fold)** | **Observed (µM*hr)** | **Simulated (µM*hr)** | **Sim/Obs**  **(fold)** |
| CE01-116 | 1 | IV 400mg | 5.4 | 6.2 | 1.13 | 10.4 | 11.8 | 1.14 |
| CE01-102 | 1 | PO 400mg | 0.7 | 0.7 | 0.96 | 5.7 | 5.1 | 0.89 |
|  | 7 | PO 400mg | 1.3 | 1.3 | 0.98 | 15.7 | 12.9 | 0.82 |
| CE01-113 | 1 | PO 800mg | 1.3 | 1.3 | 1.03 | 14.4 | 12.7 | 0.88 |
|  | 5 | PO 400mg | 1.1 | 1.3 | 1.12 | 14.8 | 12.9 | 0.87 |
| CE01-118 | 1 | IV 400mg^*^ | 1.7 | 6.1 | 3.60 | 6.8 | 11.8 | 1.74 |
|  | 3 | IV 400mg^*^ | 3.1 | 6.7 | 2.12 | 12.8 | 19.4 | 1.52 |
|  | 4 | PO 800mg | 2.0 | 2.5 | 1.23 | 23.6 | 25.2 | 1.07 |
|  | 7 | PO 400mg | 1.3 | 1.3 | 1.00 | 14.1 | 12.9 | 0.92 |

Table A3. Parameters used in the DILIsym PBPK sub-model for erythromycin.

| Parameter | Unit | Value | Source |
| --- | --- | --- | --- |
| Compound W absorption from gut | 1/hour | 1.665 | Mather 1981 (Mather et al., 1981) |
| Compound W rate of elimination in feces | 1/hour | 5.11 x 10^-5^ | Optimized |
| Compound W mol to mg | mg/mol | 733.93 | DrugBank |
| Compound W gut:blood | dimensionless | 12.0 | Optimized |
| Compound W muscle:blood | dimensionless | 0.06 | Krasniqi 2012 (Krasniqi et al., 2012) |
| Compound W other:blood | dimensionless | 22.4 | Optimized |
| Compound W liver:blood | dimensionless | 1130 | Optimized |
| Compound W active liver uptake Vmax | mg/hour/kg^0.75^ | 10.8 | Optimized |
| Compound W active liver uptake Km | mg/mL | 9.69 x 10^-3^ | Lancaster 2012 (Lancaster et al., 2012) |
| Compound W membrane permeability | mL/hour/kg^0.75^ | 1.343 | Optimized |
| Compound W fraction unbound plasma | dimensionless | 0.69 | Optimized |
| Compound W blood to plasma | dimensionless | 2.3 | Mather 1981 (Mather et al., 1981) |
| Compound W biliary clearance Vmax | mg/hour/kg^0.75^ | 0.116 | Optimized |
| Compound W renal clearance | mL/hour/kg^0.75^ | 39.4 | Optimized |
| Vmax (Compound W metabolite A) | mol/hour/kg^0.75^ | 5.37 x 10^-2^ | Riley 1997 (Riley and Howbrook, 1997) |
| Km (Compound W metabolite A) | mol/mL | 3.3 x 10^-8^ | Riley 1997 (Riley and Howbrook, 1997) |

Table A4. Comparison between observed and simulated PK parameters for erythromycin. The protocol used was the 500 mg QID protocol reported in Krasniqi 2012 (Krasniqi et al., 2012).

| Day | Sim/Obs | |
| --- | --- | --- |
|  | **C_max_** | **AUC** |
| Day 1 | 1.07 | 1.05 |
| Day 3 | 0.99 | 0.97 |

Table A5. Parameters used in the DILIsym PBPK sub-model for telithromycin.

| Parameter | Unit | Value | Source |
| --- | --- | --- | --- |
| Compound W absorption from gut | 1/hour | 0.971 | Optimized |
| Compound W rate of elimination in feces | 1/hour | 1.386 x 10^-9^ | Optimized |
| Compound W molecular weight | g/mol | 812 | DrugBank |
| Compound W gut:blood | dimensionless | 3.455 | Vieira 2012 (Vieira et al., 2012) |
| Compound W muscle:blood | dimensionless | 1.994 | Vieira 2012 (Vieira et al., 2012) |
| Compound W other:blood | dimensionless | 2.777 | Vieira 2012 (Vieira et al., 2012) |
| Compound W liver:blood | dimensionless | 2.318 | Vieira 2012 (Vieira et al., 2012) |
| Compound W fraction unbound plasma | dimensionless | 0.727 | Optimized* |
| Compound W blood to plasma | dimensionless | 0.7 | Vieira 2012 (Vieira et al., 2012) |
| Compound W biliary clearance Vmax | mg/hour/kg^0.75^ | 9.9311 | Optimized* |
| Compound W biliary clearance Km | mg/mL | 0.0434 | Vieira 2012 (Vieira et al., 2012) |
| Compound W renal clearance | mL/hour/kg^0.75^ | 545.4 | Optimized* |
| Compound W gut efflux Vmax | mg/hour/kg^0.75^ | 0.0011 | Optimized |
| Compound W gut efflux Km | mg/mL | 0.0434 | Vieira 2012 (Vieira et al., 2012) |
| Vmax (Compound W metabolite A) | mol/hour/kg^0.75^ | 3.703 x 10^-4^ | Optimized |
| Km (Compound W metabolite A) | mol/mL | 1.766 x 10^-7^ | Optimized |
| Compound W delay time constant (met A induction) | 1/hour | 0.01 | Optimized |
| Compound W metabolite A induction Vmax | 1/hour | -1 | Vieira 2012 (Vieira et al., 2012) |
| Compound W metabolite A induction Km | mg/mL | 8.817 x 10^-5^ | Vieira 2012 (Vieira et al., 2012) |
| Compound W metabolite A induction Hill | dimensionless | 6.325 | Optimized |
| Vmax (Compound W metabolite B) | mol/hour/kg^0.75^ | 3.703 x 10^-4^ | Optimized** |
| Km (Compound W metabolite B) | mol/mL | 1.766 x 10^-7^ | Optimized** |
| Compound W metabolite A biliary excretion Vmax | mg/hour/kg^0.75^ | 2.2614 | Optimized |
| Compound W metabolite A fraction unbound plasma | dimensionless | 0.056 | Optimized |
| Compound W metabolite A blood to plasma | dimensionless | 0.7 | Assumed from parent value |
| Compound W metabolite A liver to blood | dimensionless | 2.318 | Assumed from parent value |
| Compound W metabolite A renal clearance | mL/hour/kg^0.75^ | 0.246 | Optimized |
| Compound W metabolite A volume of distribution per weight | mL/kg | 2257 | Optimized |
| Compound W metabolite B biliary excretion Vmax | mg/hour/kg^0.75^ | 2.3577 | Optimized |
| Compound W metabolite B fraction unbound plasma | dimensionless | 0.056 | Optimized |
| Compound W metabolite B blood to plasma | dimensionless | 0.7 | Assumed from parent value |
| Compound W metabolite B liver to blood | dimensionless | 0.0107 | Optimized |
| Compound W metabolite B renal clearance | mL/hour/kg^0.75^ | 293036.1 | Optimized |
| Compound W metabolite B volume of distribution per weight | mL/kg | 40800 | Optimized |

Table A6. Observed (Namour et al., 2001) and simulated pharmacokinetic parameters for telithromycin for 800 mg QD dosing.

| **Day** | **Parent Sim/Obs** | | **Metabolite Sim/Obs** | | **Urinary clearance Sim/Obs** | **Fecal clearance Sim/Obs** |
| --- | --- | --- | --- | --- | --- | --- |
|  | **Cmax** | **AUC** | **Cmax** | **AUC** |  |  |
| Day 1 | 0.84 | 0.96 | 0.92 | 1.03 | 0.84 | 0.90 |
| Day 8 | 1.08 | 0.91 | 1.00 | 0.90 | N/A | N/A |

Table A7. Parameters used in the DILIsym PBPK sub-model for clarithromycin.

| Parameter | Unit | Value | Source |
| --- | --- | --- | --- |
| Kab | 1/hour | 0.83 | Optimization |
| Compound W rate of elimination in feces | 1/hour | 0.103 | Optimization |
| Compound W molecular weight | g/mol | 747.95 | - |
| Compound W blood to plasma | dimensionless | 1 | Assumed |
| Compound W fraction unbound plasma | dimensionless | 0.28 | Quinney 2010 (Quinney et al., 2010) |
| Compound W gut to blood | dimensionless | 3.52 | Optimization (R&R estimation 6.6) |
| Compound W liver to blood | dimensionless | 6.18 | Optimization (R&R estimation 11.6) |
| Compound W muscle to blood | dimensionless | 2.27 | Optimization (R&R estimation 4.3) |
| Compound W other tissue to blood | dimensionless | 1.53 | Optimization (R&R estimation 2.9) |
| Compound W renal clearance | mL/hour/kg^0.75 | 1650 | Optimized to recapitulate clinically observed urinary recovery  (Chu et al., 1992b) |
| Compound W biliary excretion Vmax | mg/hour/kg^0.75 | 0 | Davey 1991 (Davey, 1991) |
| Compound W biliary excretion Km | mg/mL | 1.00E+10 | Davey 1991 (Davey, 1991) |
| Km(Compound W metabolite A) | mol/mL | 8.00E-07 | Optimization (measured 3.5E-07) |
| Vmax(Compound W metabolite A) | mol/hour/kg^0.75 | 0.0024 | Optimization |
| Compound W delay time constant  (metabolite A induction) | 1/hour | 0.0004 | Optimization |
| Compound W metabolite A induction Vmax | 1/hour | -0.5 | Optimization |
| Compound W metabolite A induction Km | mg/mL | 4E-05 | Optimization |
| Compound W metabolite A induction Hill | dimensionless | 30 | Optimization |

Table A8. Observed (Chu et al., 1993, 1992a) and simulated pharmacokinetic parameters for a single dose and multiple doses of clarithromycin.

| Dose | Sim/Obs | |
| --- | --- | --- |
|  | **C_max_** | **AUC** |
| IV 250 mg | 1.04 | 1.05 |
| PO 250 mg (Day 1) | 1.17 | 1.36 |
| PO 250 mg (Day 4) | 1.22 | 1.66 |
| PO 500 mg (Day 1) | 0.85 | 0.84 |
| PO 500 mg (Day 4) | 0.97 | 1.09 |

Table A9. DILIsym PBPK parameters used for the representation of azithromycin.

| DILIsym Parameter | Unit | Value | Source |
| --- | --- | --- | --- |
| Compound W absorption from gut | 1/hour | 0.952 | Optimized |
| Compound W rate of elimination in feces | 1/hour | 0.124 | Optimized |
| Compound W molecular weight | g/mol | 748.996 | DrugBank |
| Compound W gut:blood | dimensionless | 1 | Optimized* |
| Compound W muscle:blood | dimensionless | 0.07 | Matzneller 2013  (Matzneller et al., 2013) |
| Compound W other:blood | dimensionless | 1 | Optimized* |
| Compound W liver:blood | dimensionless | 99.66 | Optimized* |
| Compound W fraction unbound plasma | dimensionless | 0.746 | Literature |
| Compound W blood to plasma | dimensionless | 1.544 | DrugBank |
| Compound W biliary clearance Vmax | mg/hour/kg^0.75^ | 2877 | Optimized |
| Compound W biliary clearance Km | mg/mL | 1 | Assumed linear |
| Compound W renal clearance | mL/hour/kg^0.75^ | 3221 | Optimized |
| Vmax (Compound W metabolite A) | mol/hour/kg^0.75^ | 0.0039 | Optimized |
| Km (Compound W metabolite A) | mol/mL | 1 x 10^-6^ | Assumed |

Table A10. Comparison between observed (Matzneller et al., 2013) and simulated PK parameters for 500 mg QD azithromycin.

| Day | Parent Sim/Obs | |
| --- | --- | --- |
|  | **C_max_** | **AUC** |
| Day 1 | 0.91 | 1.10 |
| Day 3 | 0.98 | 0.96 |

**Supplement B**

*In vitro assays to evaluate mitochondrial dysfunction*

To assess the potential mitochondrial signals for five macrolides, cellular respiration assays were conducted using a Seahorse XFe96 Flux Analyzer. HepG2 cells were incubated with solithromycin (0.1 – 40 µM), erythromycin (0.3 – 100 µM), telithromycin (0.1 – 75 µM), clarithromycin (0.3 – 100 µM), or azithromycin (0.1 – 40 µM) for 1 or 24 hours, and tested for an effect on cellular respiration as measured by the oxygen consumption rate (OCR). Two independent studies were conducted in triplicate. Importantly, the one hour culture was conducted in the absence of serum (*i.e.*, no serum protein binding) as is standard for the Seahorse Analyzer assay conditions. However, the 24 hour culture was too long for robust cell culture in the absence of serum. Serum was present up to 1 hour before the assay, when cell culture media was exchanged for the standard assay media. Identical parallel cultures were established to assess the intracellular concentrations of macrolides.

*In vitro assays to evaluate oxidative stress*

Induction of oxidative stress was assessed by high content screening. Briefly, HepG2 cells were plated onto black, clear bottom 96-well plates, and incubated with solithromycin (0.01 – 40 µM), erythromycin (0.03 – 100 µM), telithromycin (0.025 – 75 µM), clarithromycin (0.03 – 100 µM), or azithromycin (0.01 – 40 µM) for 1 or 24 hours. At the end of the incubation period, dihydroethidium (DHE, for ROS formation) was added, and the plate was scanned using an automated fluorescent cellular image, ArrayScan® (Thermo Scientific Cellomics). The cellular ATP content was measured using CellTiter-Glo^®^ (Promega). Two independent studies were conducted in triplicate. Identical parallel cultures were established to assess the intracellular concentrations of macrolides.

*Assessment of intracellular concentrations of macrolides*

To assess the intracellular concentration of macrolides, identical HepG2 culture was established in parallel to oxidative stress and mitochondrial assays, and cell lysate concentrations were measured by LC/MS/MS analysis. Intracellular concentrations of macrolides were calculated by correcting for cell lysate volume (100 µL) and the volume of HepG2 cells in each well, which was calculated by multiplying cell count per well by cellular volume per cell (2.85 pl/cell). Non-specific binding to the culture plates was corrected by subtracting macrolide concentrations in the no-cell plates from those in cell lysates.

*In vitro assays to evaluate bile acid transporter inhibition*

In vitro transporter inhibition assays were performed with selected macrolides, for which bile acid transporter inhibition data were not available from the published literature. The ability of drugs to inhibit the BSEP-mediated transport (and accumulation) of taurocholate was tested at 7 concentrations using membrane vesicles prepared from Sf9 cells overexpressing BSEP. MRP4 inhibition assays were performed with membrane vesicles prepared from HEK293 cells overexpressing MRP4 using dehydroepiandrosterone (DHEAS) as a probe substrate. To assess the functional impact of a drug on the human SLC (uptake) transporter NTCP, *in vitro* experiments were performed using CHO cells stably expressing NTCP. The ability of drugs to inhibit NTCP‑mediated uptake (and accumulation) of taurocholate was tested at 7 concentrations in the absence or presence of sodium. IC_50_ values were estimated by fitting a three-parameter logistic model to relative activity vs. drug concentration plot by non-linear regression.

**Supplement C**

Figure C1. The chemical structures of the five macrolide antibiotics investigated for this work.
